# The composition of the pulmonary microbiota in sarcoidosis – an observational study

**DOI:** 10.1186/s12931-019-1013-2

**Published:** 2019-02-28

**Authors:** André Becker, Giovanna Vella, Valentina Galata, Katharina Rentz, Christoph Beisswenger, Christian Herr, Jörn Walter, Sascha Tierling, Hortense Slevogt, Andreas Keller, Robert Bals

**Affiliations:** 1 Department of Internal Medicine V – Pulmonology, Allergology, Critical Care Medicine, Saarland University Hospital, Homburg, Germany; 2 Chair for Clinical Bioinformatics, Saarland University, Saarbrücken, Germany; 3 Department of Genetics/Epigenetics, Faculty NT, Saarland University, Saarbrücken, Germany; 4 Septomics Research Center, Jena University Hospital, Jena, Germany

**Keywords:** Microbiome, Sarcoidosis, Interstitial lung disease, Inflammation, Infection

## Abstract

**Background:**

Sarcoidosis is a systemic disease of unknown etiology. The disease mechanisms are largely speculative and may include the role microbial patterns that initiate and drive an underlying immune process. The aim of this study was to characterize the microbiota of the lung of patients with sarcoidosis and compare its composition and diversity with the results from patients with other interstitial lung disease (ILD) and historic healthy controls.

**Methods:**

Patients (sarcoidosis, *n* = 31; interstitial lung disease, *n* = 19) were recruited within the PULMOHOM study, a prospective cohort study to characterize inflammatory processes in pulmonary diseases. Bronchoscopy of the middle lobe or the lingula was performed and the recovered fluid was immediately sent for analysis of the pulmonary microbiota by 16sRNA gene sequencing. Subsequent bioinformatic analysis was performed to compare the groups.

**Results:**

There were no significant differences between patients with sarcoidosis or other ILDs with regard to microbiome composition and diversity. In addition, the abundance of the genera *Atopobium*, *Fusobacterium*, *Mycobacterium* or *Propionibacterium* were not different between the two groups. There were no gross differences to historical healthy controls.

**Conclusion:**

The analysis of the pulmonary microbiota based on 16sRNA gene sequencing did not show a significant dysbiosis in patients with sarcoidosis as compared to other ILD patients. These data do not exclude a microbiological component in the pathogenesis of sarcoidosis.

**Electronic supplementary material:**

The online version of this article (10.1186/s12931-019-1013-2) contains supplementary material, which is available to authorized users.

## Background

Sarcoidosis is a systemic disease that is characterized by typical histopathology with noncaseating granuloma formation in affected organs. While most organs can be affected, the pulmonary manifestation is the most frequent manifestation. The clinical presentation is heterogenous, what makes diagnostic procedures often difficult. This heterogeneity also points toward complex and possible multiple disease mechanisms. The pathomechanisms of sarcoidosis are still not entirely clear but involve antigen-driven CD4+ T-cell activation with macrophage attraction and granuloma formation. There are several hypotheses about the causative antigen that initiates and drives the underlying immune process. Some studies indicated that autoimmunity may play a role in these processes [1, 2]. A potential microbial compound has been discussed in the last decades. While classical culture-dependent microbiology did not unravel a clear candidate, remnant microbial molecules were identified in sarcoidosis patients, many data sets point towards the involvement of mycobacterial species [2–4]. In addition, also other microbial classes such as *Haemophilus*, *Atopobium* and *Fusobacterium* may show alterations in their abundance in sarcoidosis [5, 6].

The development of technologies to characterize the microbiota of a given habitat offers opportunities to further understand the role of microorganisms in disease development. Microbiome analysis is based on the analysis of microbial genes or transcripts. Current techniques use 16sRNA gene sequencing or whole genome shot-gun sequencing with subsequent bioinformatic analysis. Only a few studies have been performed in sarcoidosis and suggested that there are no or little differences in the microbial composition between sarcoidosis and control groups [6–8]. In addition, a few studies have also characterized the microbiota in other interstitial lung disease (ILD) [9, 10].

The aim of this study was to characterize the microbiota in the lungs of patients with sarcoidosis and to compare these data with results from other interstitial lung diseases and historical healthy controls. The study aimed to show whether there is unique phenotype of the sarcoidosis microbiome as compared to a heterogenous group of other ILDs. We performed analysis of the microbiome by 16sRNA-gene sequencing and biostatistical analysis.

## Methods

### Study population

Patients were recruited within the PULMOHOM study, a prospective cohort study to characterize inflammatory processes in pulmonary diseases. Patients with suspected sarcoidosis or interstitial lung diseases (ILD) undergoing bronchoscopy participated in the study. Patient were selected based on clinical and radiological finding indicating high probability for these disease entities.

Patients were further diagnosed by standard diagnostic procedures including bronchoalveolar lavage, biopsy, CT scan and serological markers. The final diagnosis was used to segregate the patients in the diagnostic groups (sarcoidosis, ILD).

The PULMOHOM study was approved by the ethics board of the Landesärztekammer des Saarlandes and informed written consent was obtained from all patients.

### Bronchoalveolar lavage (BAL) and processing

Bronchoscopy was performed as described earlier [11] and BAL of a segment of the middle lobe or the lingula was performed by instillation of 5 times 20 mL of sterile saline and recovered fluid was immediately sent for further analysis.

### DNA isolation and 16sRNA gene sequencing

PCRs of V1/V2 and V3/V4 variable regions of 16S rRNA genes were performed (5–25 ng template, 80 mM Tris-HCL, 20 mM (NH4)2SO4, 0.2% Tween-20, 2.5 mM MgCl2, 0.2 mM of each dNTP, 2.5 U HotFirePol (Solis BioDyne)) using 200 pmol of each primer (V1/V2: forw, 5′-agagtttgatcctggctcag-3′ and rev, 5′-tgctgcctcccgtaggagt-3′; V3/V4: forw, 5′-cctacgggnggcwgcag-3′ and rev, 5′-gactachvgggtatctaatcc-3′) with Illumina universal TruSeq adaptor sequences attached at the 5′-end. PCRs were performed in a thermocycler starting with 15 min 95 °C followed by 35 cycles 95 °C 1 min, 54 °C 1 min, 72 °C 1 min and a 5 min final extension at 72 °C. Amplicons were purified with MagSi-NGS PREP Plus beads (Steinbrenner, Wiesenbach, Germany), diluted, pooled and sequenced on the Illumina MiSeq (v3 chemistry: 2 × 300 bp paired-end) following the manufacturer’s instructions aiming at 50,000 reads per sample.

### Biostatistical analysis of microbiome data

Read quality reports were generated from the FASTQ files using FastQC (version 0.11.7) and a summary was created using MultiQC (version 1.6). Operational taxonomic units (OTUs) were defined for both amplicons separately using LotuS (version 1.565). OTUs are clusters of sequences grouped together based on sequence similarity; one sequence is chosen to represent the complete cluster [12]. All obtained OTU sequences were additionally searched in the BLAST nr/nt database (created on 04.08.2014) using BLASTn (version 2.6.0+) with minimal query coverage per HSP and identity cutoff of 80% and by saving maximal 10 hits per query. For each OTU, the best hit was kept by sorting the associated hits by query coverage per HSP and percentage identity. For each of the remaining hits, the subject accession ID was extracted and searched in the NCBI database to retrieve the assigned taxon ID using EDirect (version 9.50). The taxon ID was searched in the NCBI taxonomy database to get the associated taxonomic lineage. For each amplicon, the OTUs were filtered to remove likely contaminants. The OTUs were flagged if they had “Eukaryota” in the superkingdom taxon name derived from the BLAST hits, or if they were present in the NTC sample, or if they had a count below 1 in all non-control samples (i.e. all samples except the NTC sample). All flagged OTUs were then removed from the OTU count table of the non-control samples. An overview of the taxonomic composition of all kept OTUs was created per amplicon over all samples and for each sample indication group using Krona (version 2.5). In the following, only OTUs kept after the filtering step were considered and the analysis were performed for both amplicons (V1/V2 and V3/V4) separately.

The R-package “ampvis2” (version 2.3.2) was used to plot rarefaction curves (number of reads vs. the number of observed OTUs, step size 500), and to compute and plot the alpha-diversity values for all provided measures (i.e. “observed”, “shannon”, “simpson”, and “invsimpson”). The alpha-diversity values between the indication groups were compared using the Wilcox test (two-sided, confidence level 0.95) from the R-package coin (version 1.2–2).

For CLR-transformation, zero imputation was applied to the OTU count matrix using the method “cmultRepl” from the R-package “zCompositions” (version 1.1.1, parameters: method = “CZM”, label = 0, output = “prop”). Then, the sample values were CLR-transformed using the method “clr” from the R-package “compositions” (version 1.40–1). Principal component analysis (PCA) was applied to the resulting matrix (method “prcomp”, parameters: center = TRUE, scale. = FALSE). Also, a heatmap was created using hierarchical clustering (Euclidean distance, complete linkage) to re-order the samples and OTUs.

For the genera *Atopobium, Fusobacterium, Mycobacterium and Propionibacterium*, the corresponding OTUs were identified (based on the taxonomy assigned by LotuS). A heatmap showing only the CLR-transformed counts of these OTU was plotted and the samples were re-ordered using hierarchical clustering (Euclidean distance, complete linkage).

The R-package “ALDEx2” (version 1.8.0) was used for differential analysis of the OTU counts by comparing the indication groups. The counts were first CLR-transformed using the “aldex.clr” method (parameter: denom = “iqlr”). Then, the effect sizes were calculated using the method “aldex.effect”: Finally, the Wilcoxon test was applied.

The code used for the analysis can be found at https://github.com/VGalata/16S_Sarcoidosis. The original sequencing data are available at https://www.ncbi.nlm.nih.gov/sra/PRJNA510549.

## Results

### Patient characterization

The characteristics of the study population are presented in Table 1. Based on the radiologic evaluation, patients were classified into the Scadding group: 0 (1), I (10), II (13), III (4) and IV (3). As compared to the ILD control groups, patients with sarcoidosis were significantly younger and had a lower FEV1/VC ratio. The number of lymphocytes in the BAL was significantly elevated and the CD4/CD8 ratio was increased. The group of ILD patients comprised individuals with rheumatoid-related ILD (*n* = 2), sclerodermia-associated ILD (*n* = 1), drug-related ILD (*n* = 3), hypersensitivity pneumonitis (n = 2), idiopathic pulmonary fibrosis (n = 3), nonspecific interstitial pneumonia (NSIP, *n* = 7), and cryptogenic organizing pneumonia (COP, n = 1).

**Table 1:** Patient characteristics

Clinical characteristics	Sarcoidosis	Interstitial lung disease	p-value
Number of subjects, n (%)	31 (62%)	19 (38%)	–
Female, n (%)	14 (45%)	8 (42%)	–
Male, n (%)	17 (55%)	11 (58%)	–
Age (years), median (range)	51 (22–79)	66 (57–82)	0.0003
Smoking			
Non-smokers, n (%)	18 (58%)	6 (31.5%)	0.06
Current-smokers, n (%)	7 (22.5%)	2 (10.5%)	0.25
Former-smokers, n (%)	6 (19.5%)	9 (47%)	0.05
BMI, median (range)	27 (17–37.1)	24.7 (16.8–34.9)	0.5
Pulmonary function test			
FEV1%, median (range)	81.5 (61.5–126.8)	66.8 (49–109.6)	0.66
FEV1/VC, median (range)	79.8 (66.2–86.5)	85.7 (61.4–108.8)	0.02
TLC (l), median (range)	5.54 (4.17–8.38)	4.2 (2.56–7.99)	0.26
ERV (l), median (range)	0.78 (0.21–1.39)	0.55 (0.12–1.97)	0.89
RV (l), median (range)	2.12 (1.12–4.54)	1.76 (1.41–2.87)	0.60
VC (l), median (range)	3.34 (1.9–5.61)	2.34 (0.88–5.6)	0.26
Bronchoscopy, BAL			
BAL recovery %, median	50	55	
Neutrophils %, median	9	14	0.05
Macrophages %, median	49	62	0.01
Lymphocytes %, median	38	25.5	0.02
Eosinophils %, median	1	2	0.19
Basophils %, median	0	0	
CD4/CD8 ratio, median	3.2	0.8	0.03
Laboratory			
CRP mg/dl, median (range)	4.2 (0.4–83.9)	5.4 (0.6–326.3)	0.44
Leukocytes (1/μl), median (range)	5.65 (3–13.8)	7.55 (3.4–13.9) 0.12	

### Microbiome features of sarcoidosis and ILD patients

**Fig. 1:**
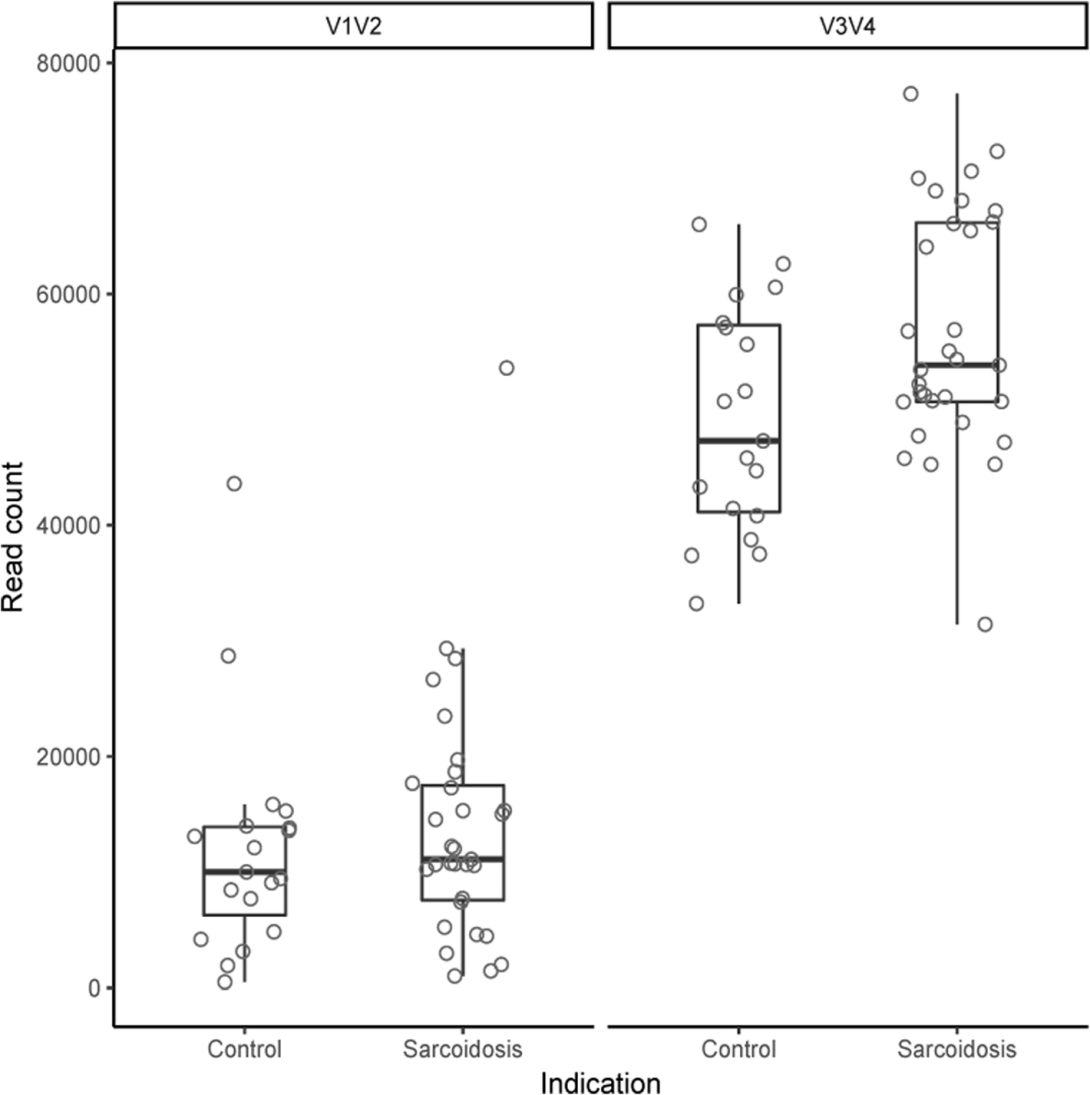
Number of raw reads per sample for V1/V2 and V3/V4. The samples were grouped by their indication (“Control” and “Sarcoidosis”). The distribution of the read counts is shown by boxplots

16sRNA gene sequencing was applied to characterize the lung microbiota of 31 patients with sarcoidosis and 19 with other interstitial lung diseases. The median number of reads was 10,951 (control: 10,023; sarcoidosis: 11,118) for V1/V2 and 51,916 (control: 47,312; sarcoidosis: 53,879) for V3/V4, respectively (Fig. 1). In total, 1212 (V1/V2) and 1743 (V3/V4) OTUs were defined. The original sequencing data are available at https://www.ncbi.nlm.nih.gov/sra/PRJNA510549.

The percentage of counts belonging to OTUs removed during the filtering step was variable. While these percentage values were below 40% for most of the V1/V2 samples, in the V3/V4 samples, the fraction of counts was above 75% for more than half of the samples (Additional file 1: Figure S1 A and B). The largest fraction of OTU counts in V1/V2 samples belonged to OTUs present in the control (NTC) sample. In the V3/V4 samples, however, the largest fraction belonged to OTUs present in the NTC sample and was classified as contamination (based on the BLAST hits). In the phylogenetic tree of all OTUs, most removed OTUs were classified as contaminants (Additional file 1: Figure S2 A and B). Also, most of these OTUs were placed in a separate subtree distant from the remaining OTUs. The V3/V4 samples demonstrated a higher frequency of OTUs flagged as contaminants than the V1/V2 samples.

### Composition of the microbiota

**Fig. 2:**
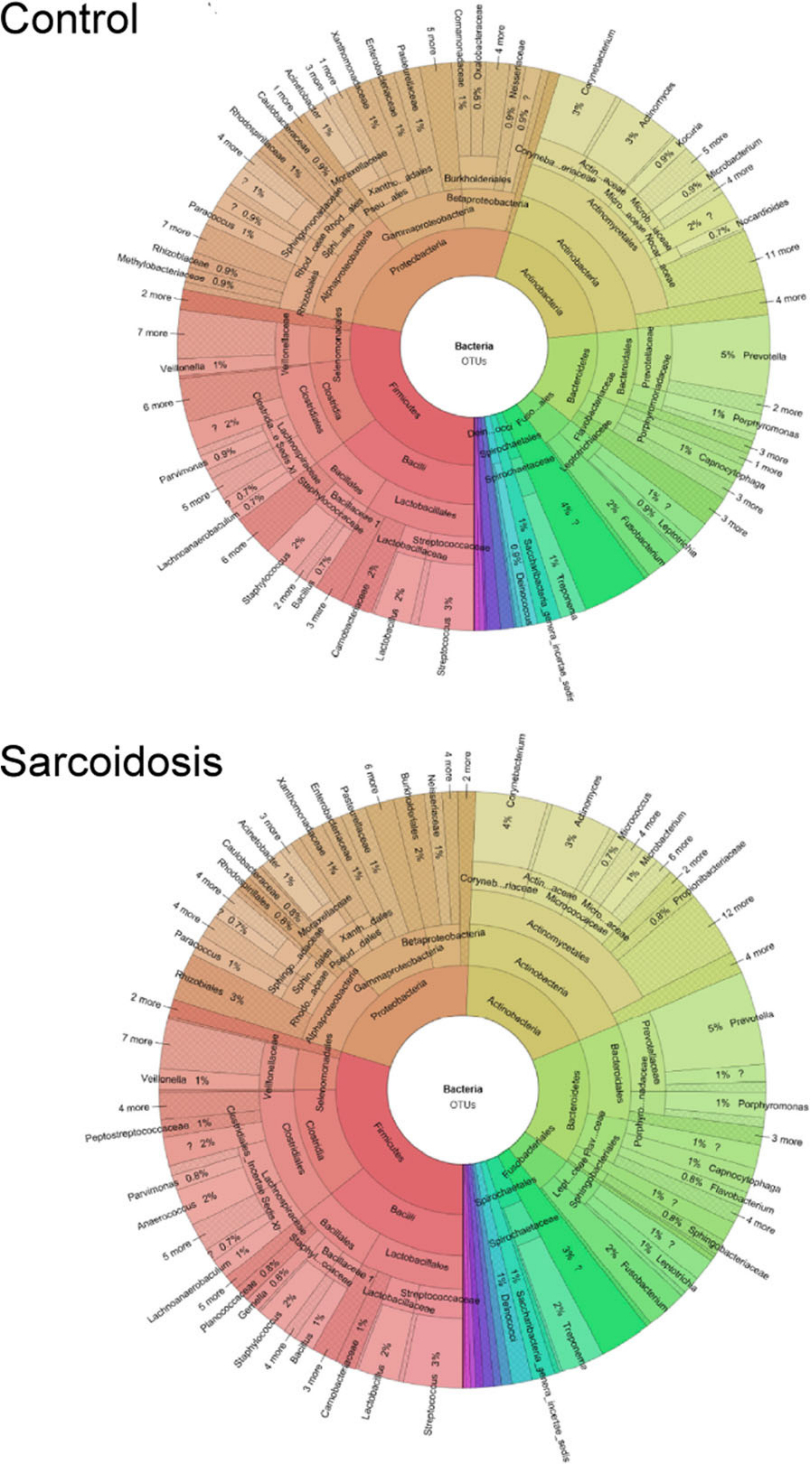
Taxonomic composition of the dataset based on taxa assigned by LotuS. Only OTUs not removed during the filtering process were considered

The taxonomic composition of the remaining OTUs is illustrated in Fig. 2 (the interactive version of the plot can be found in the online supplement). In total, 902 and 685 OTUs remained after the filtering step in the V1/V2 and V3/V4 datasets, respectively. For both amplicons, a similar distribution of the most frequent genera was found. In V1/V2, 231 unique genera were found where four taxa had a frequency of above 2%: *Prevotella* (37 OTUs, 4.1%), *Corynebacterium* (28 OTUs, 3.1%), *Actinomyces* (25 OTUs, 2.77%), and *Streptococcus* (24 OTUs, 2.66%). In V3/V4, 194 unique genera were observed with 5 taxa having a frequency above 2%: *Prevotella* (46 OTUs, 6.72%), *Actinomyces* (33 OTUs, 4.82%), *Streptococcus* (31 OTUs, 4.53%), *Treponema* (19 OTUs, 2.77%), and *Corynebacterium* (15 OTUs, 2.19%). In 232 (25.72%) and 146 (21.31%) cases the genus was unknown in the V1/V2 and V3/V4 datasets, respectively. There was no significant difference between patients with sarcoidosis and ILD.

### Richness and diversity

**Fig. 3:**
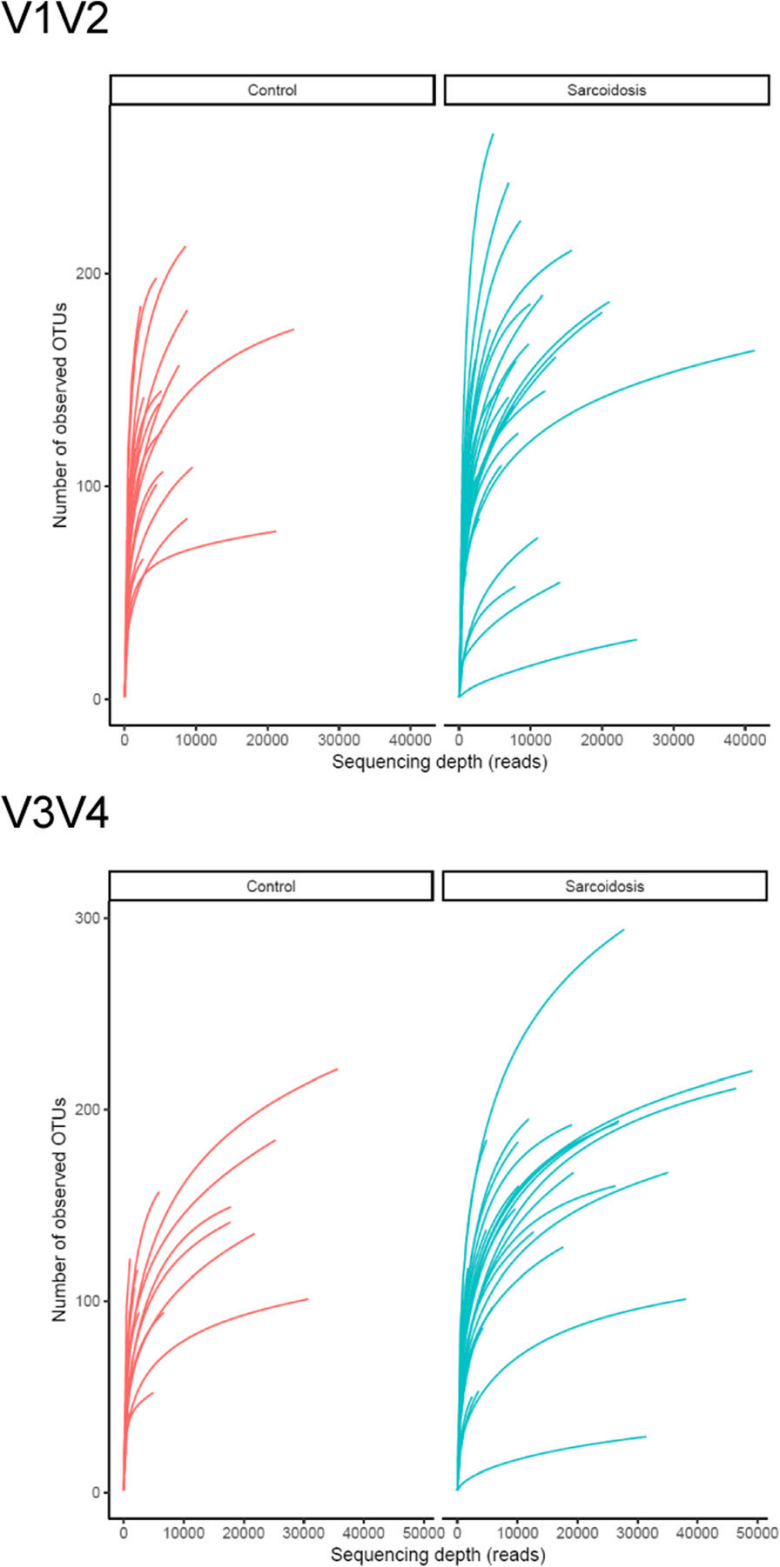
Rarefaction curves (**a**) for V1/V2 and (**b**) for V3/V4, and alpha-diversity (**c**) for V1/V2 and (**d**) for V3/V4. The rarefaction curves were plotted for both sample groups separately. The alpha-diversity plot shows the distribution (boxplot) and individual sample values (circles), and whether the distributions were found significantly different between the sample groups (“NS” means not significant)

Rarefaction analysis was performed and demonstrated no trend of lower richness (number of OTUs) in one of the groups (Fig. 3 a and b). For only some samples, the curve showed a saturation shape, i.e. the richness most of these samples cannot be considered as completely sampled. For both amplicons and all tested alpha-diversity measures, the alpha-diversity distribution was not significantly different between the two groups (two-sided Wilcoxon test, alpha = 0.05, Fig. 3 c and d).

### Unsupervised analysis of sample clusters

**Fig. 4:**
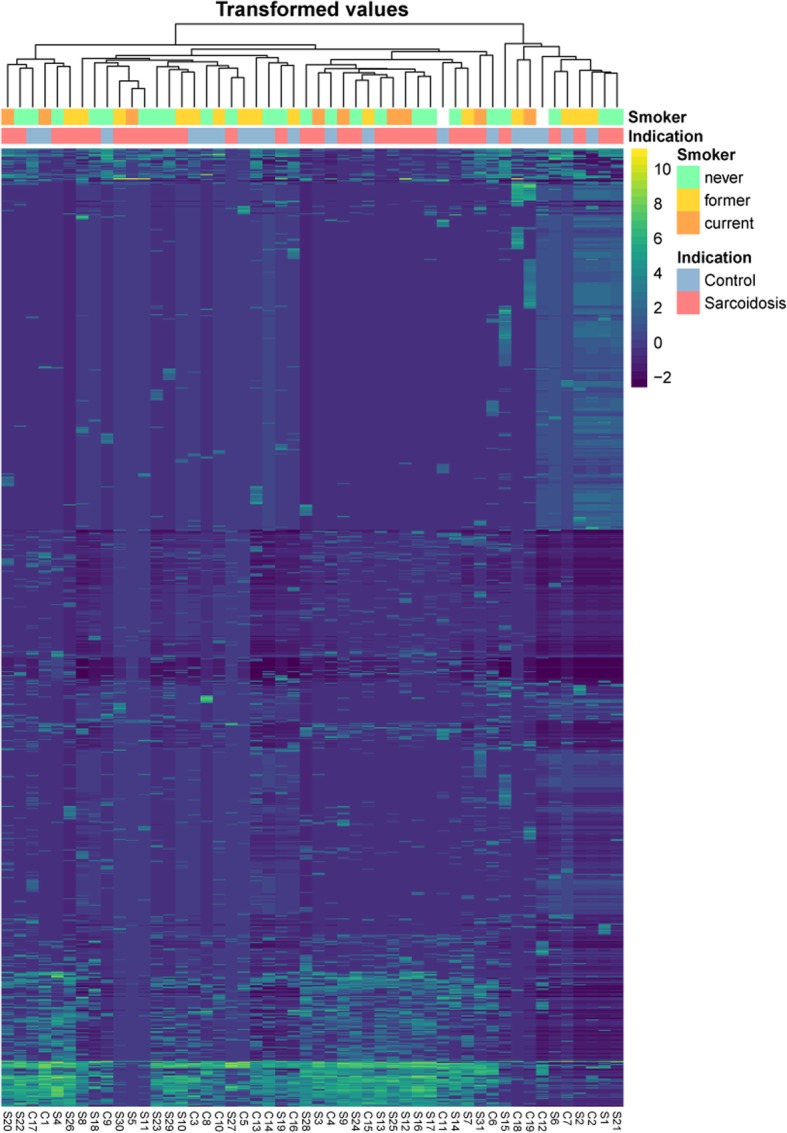
Heatmap of CLR-transformed OTU counts for V1/V2. The samples and OTUs were re-ordered by hierarchical clustering and additional annotation was added to the resulting sample tree – the sample group label (“Control” or “Sarcoidosis”) and smoker status (“never”, “former”, “current”)

The PCA analysis on the CLR-transformed OTU counts did not show any notable clusters of samples related to the disease groups or smoker status (Additional file 1: Figure S3 a and b). The variance explained by the two first principal components was approximately 37.48% for V1/V2 and 41.66% for V3/V4). The same holds for the heatmaps, where the samples were grouped by hierarchical clustering, using all OTUs (Fig. 4) and only OTUs assigned to genus *Atopobium*, *Fusobacterium*, *Mycobacterium* or *Propionibacterium* (Additional file 1: Figure S4).

### Differential analysis of OTUs

**Fig. 5:**
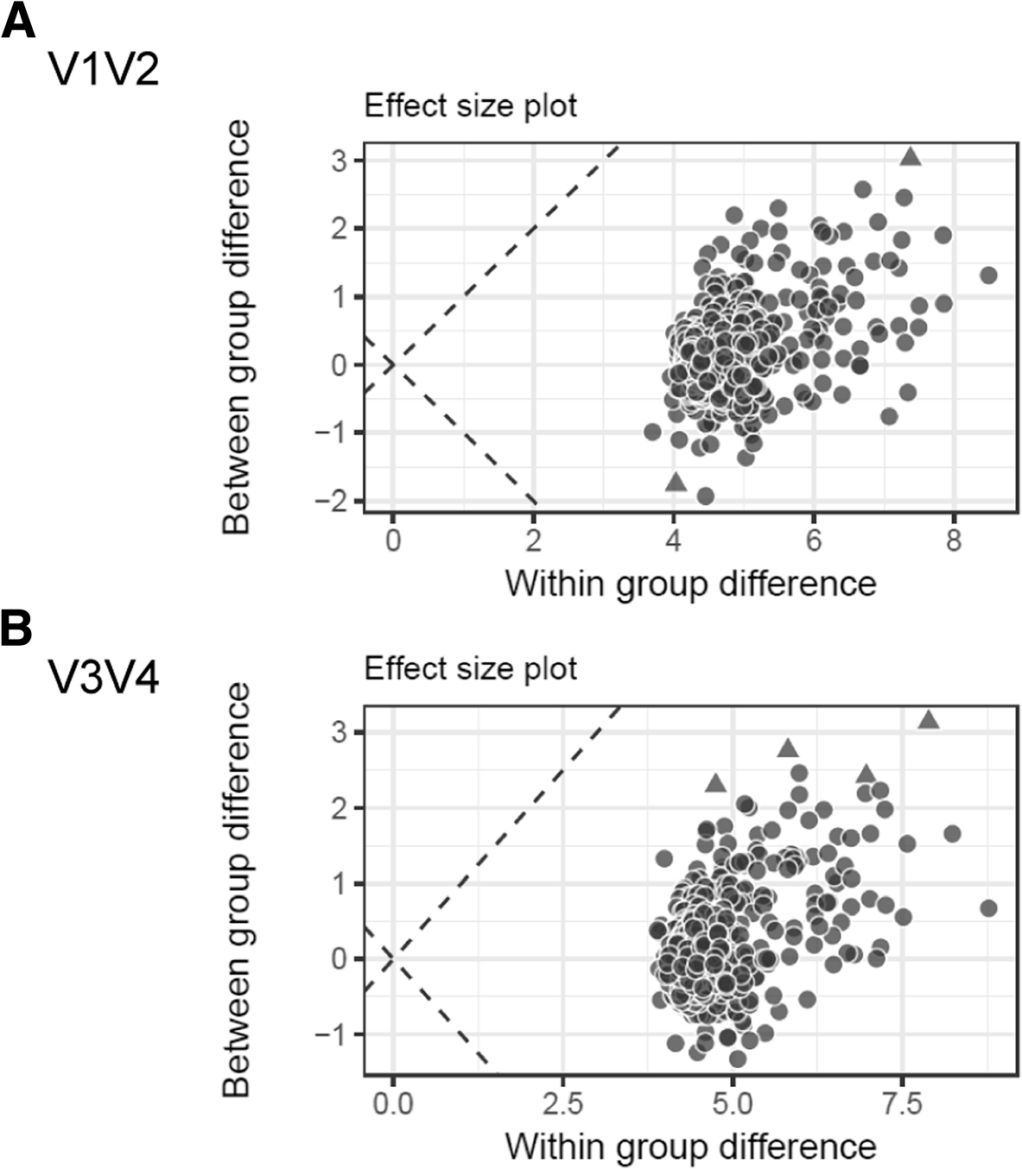
Results of differential analysis of OTUs for (**a**) V1/V2 and (**b**) V3/V4. The effect size plot shows the within and between group differences. The dashed lines correspond to points where the within and between group differences would be the same

To identify OTUs that significantly differ between the groups, a differential analysis was performed and showed that none of the OTUs had an effect size with an absolute value larger than 0.5, and only 2 and 4 OTUs had a *p*-value below 0.05 for the V1/V2 and V3/V4 amplicons, respectively: OTU_90 (*Staphylococcus*) and OTU_15 (*Actinomyces*) in V1/V2, and OTU_75 (*Mogibacterium*), OTU_20 (*Actinomyces*), OTU_220 (Saccharibacteria_genera_incertae_sedis) and OTU_22 (*Atopobium*) in V3/V4 (Fig. 5). These data indicate that there are no meaningful differences with respect to specific OTUs between sarcoidosis and other ILD in the present study.

## Discussion

The present study characterized the pulmonary microbiome of patients with sarcoidosis in comparison to patients with ILD and historical healthy controls. The main findings were that there are no significant differences between the sarcoidosis and the ILD groups with regard to the overall structure, to the diversity, and to differential analysis.

It has been hypothesized for a long time that microorganisms have a role in the development of sarcoidosis [13, 14]. While there is no evidence that sarcoidosis is an infectious disease, DNA or other components of microorganisms have been detected in sarcoidosis biosamples. There is evidence that DNA from *Propionibacterium acnes* [15], peptides or DNA [16] from *Mycobacterium* are correlated with the active sarcoidosis [3]. In addition, significantly more patients with sarcoidosis show the presence of IgG directed to recombinant mycobacterial katG in serum [17]. Which exact roles these microbial patterns could play in the pathogenesis is not clear, one view is that they could initiate the formation of persistent, granulomatous inflammation. The present study applies microbiome analysis based on 16sRNA gene sequencing to characterize the microbiota in sarcoidosis BAL samples. We found no significant difference between the numbers of OTUs, the composition or the diversity between patients with sarcoidosis and ILD. Of note, about 20% of the OTUs could not be assigned to a genus. Whether these unknown OTUs could include disease-specific species is unknown. Similar results were obtained in most other microbiome studies addressing this disease [7, 8, 18]. A study investigated different approaches to samples lung specimens, performed whole-genome sequencing and found enrichment of microbes in tissue (fungi and *Cladosporiaceae*), BAL (*Aspergillus*), however, “no microbial lineage was enriched in more than one sample type after correction for multiple comparisons” [7]. A 16sRNA gene sequencing approach was used to compare the microbiota in BAL from patients with rheumatoid arthritis, sarcoidosis and healthy controls. The microbiota of patient groups were less diverse than that from healthy controls but similar to each other [18]. One study compared the microbiota of sarcoidosis patients with those from interstitial lung disease, *Pneumocystis* pneumonia and healthy controls and found no differences in the overall composition and diversity between these groups [8].

To compare the microbiota from sarcoidosis patients with those from healthy controls we used historical data from our studies [11] and from other researchers [19, 20]. This historical approach is limited to the comparison of the gross structure of the microbiota. In the present study, the lung microbiota in sarcoidosis patients is composed of the major groups *Firmicutes*, *Proteobacteria*, *Acinetobacter*, *Bacteroidetes*, *Fusobacteriales*, and *Spirochaetales*. Healthy controls from an earlier study using T-FRLP, cloning and sequencing revealed the presence of these groups. Based on the methodological differences, a direct comparison is difficult [11]. One study in healthy individuals investigated the difference of the microbiome depending on the topology of the sampling and found a qualitatively equivalent composition of the microbiota [19]. Quantitative differences may have been caused by methodological differences. Another study with material from healthy individuals shows similar results (dominance of Bacteroides, Firmicutes, Proteobacteria, and Fusobacteria). In contrast, a recent study in 71 patients with sarcoidosis, 15 patients with idiopathic pulmonary fibrosis and 10 healthy controls found a significant difference in the redundancy analysis between the sarcoidosis and the healthy control group [6]. The genera *Atopobium* and *Fusobacterium* were found more frequently in the sarcoidosis group and also an association with the Scadding type was reported.

This and other studies on th microbiome did not unravel a disease-characteristic change of the pulmonary microbiota in the lungs of patients with sarcoidosis, while some few other studies did find differences. It is currently unclear whether this indicates that microbial patterns have no role in the development of the disease or whether the methodologies applied have limitations that preclude the repeated validation of such alterations of the microbiota.

The present study has strengths and weaknesses: One issue is the selection of the optimal source specimen for the microbiome analysis. As pathomechanisms of sarcoidosis likely takes place within the distal lung parenchyma, BAL might be a favorable material. The study of Clark and colleagues showed small but highly variable differences between sample types [7]. A larger number of patients could have allowed to associate specific changes of the microbiota with clinical phenotypes. Due to the limitations in sample size, it was not possible to discriminate patients by phenotype (Scadding groups) or disease severity. We chose to compare sarcoidosis patients with patients with interstitial lung disease because both groups share at least some pathomechanisms and we sought to identify sarcoidosis-specific changes of the microbiota. The ILD group is heterogenous and comprises several specific disease entities, which likely increases the variability of the results from this group. We could not include a healthy-control group due to restrictions imposed by the local ethics committee. The comparison between actual and historical data is difficult and likely not conclusive. The analysis of 16sRNA genes has its inherent limitations such as limited resolution and lower sensitivity as compared to shotgun metagenomic sequencing [7]. In contrast, analysis of the pulmonary microbiome is more difficult with metagenomic approaches due to the low abundance of microbial DNA.

## Conclusion

We could not detect a significant difference between the lung microbiota of patients with sarcoidosis as compared to patients with other interstitial lung diseases.

## Additional file

Additional file 1: **Figure S1.** Percentage of OTU counts per sample covered by OTUs not removed (“kept”) or discarded during the filtering step for (**A**) V1/V2 and (**B**) V3/V4. OTUs were removed if they were considered as contaminants (flag “contamination”), were present in the NTC sample (flag “controls”), had a count below 1 in non-control samples (i.e. all samples except the NTC sample; flag “count”), or any combination thereof. **Figure S2.** Phylogenetic OTU tree constructed by LotuS for (**A**) V1/V2 and (**B**) V3/V4. The OTUs were colored based on whether they were kept (flag “kept”) or discarded (flags “contamination”, “controls”, “counts”, or any combination thereof) during the filtering step. OTUs were removed if they were considered as contaminants (flag “contamination”), were present in the NTC sample (flag “controls”), or had a count below 1 in non-control samples (i.e. all samples except the NTC sample; flag “count”). **Figure S3.** PCA plots for (**A**) V1/V2 and (**B**) V3/V4. The plots show all samples using the two first principal components. The percentage of the variance explained by each principal component can be found in the axis title. The samples were colored with respect to their indication group. **Figure S4.** Heatmap of CLR-transformed OTU counts for (**A**) V1/V2 and (**B**) V3/V4 only for OTUs assigned by LotuS to the genus *Atopobium*, *Fusobacterium*, *Mycobacterium* or *Propionibacterium*. The samples were grouped by hierarchical clustering and additional annotation was added to the resulting tree – the sample group label (“Control” or “Sarcoidosis”) and smoker status (“never”, “former”, “current”). (ZIP 1170 kb)

## Abbreviations

BAL: Bronchoalveolar lavage
CLR: Centered log ratio
COPD: Cryptogenic organizing pneumonia
CT: Computer tomography
ILD: Interstitial lung disease
NSIP: Nonspecific interstitial pneumonia
OTU: Operational taxonomic unit
PCR: Polymerase chain reaction
RNA: Ribonucleic acid
