# Supplementary material for: The composition of the pulmonary microbiota in sarcoidosis – an observational study
Source: Respir Res. 2019 Feb 28;20:46. doi: 10.1186/s12931-019-1013-2 (PMC6396534; doi:10.1186/s12931-019-1013-2)
Supplement: Supplementary file 1 — Figure S1. Percentage of OTU counts per sample covered by OTUs not removed (“kept”) or discarded during the filtering step for (A) V1/V2 and (B) V3/V4. OTUs were removed if they were considered as contaminants (flag “contamination”), were present in the NTC sample (flag “controls”), had a count below 1 in non-control samples (i.e. all samples except the NTC sample; flag “count”), or any combination thereof. Figure S2. Phylogenetic OTU tree constructed by LotuS for (A) V1/V2 and (B) V3/V4. The OTUs were colored based on whether they were kept (flag “kept”) or discarded (flags “contamination”, “controls”, “counts”, or any combination thereof) during the filtering step. OTUs were removed if they were considered as contaminants (flag “contamination”), were present in the NTC sample (flag “controls”), or had a count below 1 in non-control samples (i.e. all samples except the NTC sample; flag “count”). Figure S3. PCA plots for (A) V1/V2 and (B) V3/V4. The plots show all samples using the two first principal components. The percentage of the variance explained by each principal component can be found in the axis title. The samples were colored with respect to their indication group. Figure S4. Heatmap of CLR-transformed OTU counts for (A) V1/V2 and (B) V3/V4 only for OTUs assigned by LotuS to the genus Atopobium, Fusobacterium, Mycobacterium or Propionibacterium. The samples were grouped by hierarchical clustering and additional annotation was added to the resulting tree – the sample group label (“Control” or “Sarcoidosis”) and smoker status (“never”, “former”, “current”). (ZIP 1170 kb) [file 12931_2019_1013_MOESM1_ESM.zip › Sarcoidosis Supplementary Information 2019.doc]

**SUPPLEMENTARY INFORMATION**

**The composition of the pulmonary microbiota in sarcoidosis**

André Becker^1 *^, Giovanna Vella^1 *,^ Valentina Galata^2^, Katharina Rentz^1^, Christoph Beisswenger^1^, Christian Herr^1^, Jörn Walter^3^, Sascha Tierling^3^, Hortense Slevogt^4^, Andreas Keller^2^, Robert Bals ^1^

^1^ Department of Internal Medicine V - Pulmonology, Allergology, Critical Care Medicine, Saarland University Hospital, Homburg, Germany.

^2^ Chair for Clinical Bioinformatics, Saarland University, Saarbrücken, Germany

^3^ Department of Genetics/Epigenetics, Faculty NT, Saarland University, Saarbrücken, Germany

^4^ Septomics Research Center, Jena University Hospital, Jena, Germany

**Figure S1:** Percentage of OTU counts per sample covered by OTUs not removed (“kept”) or discarded during the filtering step for (**A**) V1/V2 and (**B**) V3/V4. OTUs were removed if they were considered as contaminants (flag “contamination”), were present in the NTC sample (flag “controls”), had a count below 1 in non-control samples (i.e. all samples except the NTC sample; flag “count”), or any combination thereof.

**Figure S2:** Phylogenetic OTU tree constructed by LotuS for (**A**) V1/V2 and (**B**) V3/V4. The OTUs were colored based on whether they were kept (flag “kept”) or discarded (flags “contamination”, “controls”, “counts”, or any combination thereof) during the filtering step. OTUs were removed if they were considered as contaminants (flag “contamination”), were present in the NTC sample (flag “controls”), or had a count below 1 in non-control samples (i.e. all samples except the NTC sample; flag “count”).

**Figure S3:** PCA plots for (**A**) V1/V2 and (**B**) V3/V4. The plots show all samples using the two first principal components. The percentage of the variance explained by each principal component can be found in the axis title. The samples were colored with respect to their indication group.

**Figure S4:** Heatmap of CLR-transformed OTU counts for (**A**) V1/V2 and (**B**) V3/V4 only for OTUs assigned by LotuS to the genus *Atopobium*, *Fusobacterium*, *Mycobacterium* or *Propionibacterium*. The samples were grouped by hierarchical clustering and additional annotation was added to the resulting tree – the sample group label (“Control” or “Sarcoidosis”) and smoker status (“never”, “former”, “current”).
